# Methods of Cell Propulsion through the Local Stroma in Breast Cancer

**DOI:** 10.1155/2014/197480

**Published:** 2014-04-07

**Authors:** Kerry J. Davies

**Affiliations:** Canniesburn Plastic Surgery Unit, Glasgow Royal Infirmary, Castle Street, Glasgow G4 0SF, UK

## Abstract

In the normal breast, cellular structures change cyclically in response to ovarian hormones. Cell proliferation, apoptosis, invasion, and differentiation are integral processes that are precisely regulated. Normal epithelial cells depend on the formation of intercellular adhesion contacts to form a continuous sheet of stratifying cell layers that are attached to one and other horizontally and vertically. Cells migrate by extending membrane protrusions to explore the extracellular space locating their targets in a chemotactic manner. The formation of cell protrusions is driven by the assembly of actin filaments at the leading edge. Reorganisation is regulated by a highly integrated signalling cascade that transduces extracellular stimuli to the actin filaments. This signalling cascade is governed by GTPases which act as molecular switches leading to actin polymerisation and the formation of filopodia and lamellipodia. This process is linked to downstream molecules known collectively as WASP proteins, which, in the presence of cortactin, form a complex leading to nucleation and formation of branched filaments. In breast cancer, the cortactin is over expressed leading to increased cellular motility and invasiveness. This hugely complex and integrated signalling cascade transduces extracellular stimuli. There are multiple genes related to cell motility which are dysregulated in human breast cancers.

## 1. Introduction

Breast cancer is by far the most frequent cancer among women with an estimated 1.38 million new cancer cases diagnosed in 2008 (23% of all cancers) and ranks second overall (10.9% of all cancers) [1, 2]. Breast cancer is estimated to be responsible for around 458,500 female deaths in 2008 or nearly one in seven (around 14%) of all cancer deaths in women [3]. Cancer mortality is related to metastatic spread to other organs [4–6]. Therefore, early detection in order to improve breast cancer outcome and survival remains the cornerstone of breast cancer control [7].

The extracellular matrix (ECM) is composed of highly variable and dynamic components that regulate cell behavior. The protein composition and physical properties of the ECM govern cell fate through biochemical and biomechanical mechanisms. This requires carefully orchestrated and thorough regulation. In breast cancer, many ECM proteins are significantly deregulated and specific matrix components promote tumor progression and metastatic spread. Several ECM proteins that are associated with breast cancer development overlap substantially with a group of ECM proteins induced during the state of tissue remodeling such as mammary gland involution [8]. Understanding the regulatory role of the ECM will provide insight into mechanisms underlying normal and pathological development of the mammary gland [9].

Stromal tissue is composed of supporting cells and connective tissue, comprises a large component of the local microenvironment of many epithelial cell types, and influences several fundamental aspects of cell behaviour through both tissue interactions and niche regulation. The significance of the stroma in development and disease has been increasingly recognised [10].

The breast mesenchyme is comprised of complex connective tissue composed of heterogeneous cell types, including fibroblasts, adipocytes, immune cells, endothelial cells, pericytes, nerve cells, and acellular matrix components, such as collagen I, collagen III, collagen IV, proteoglycans, and glycoproteins [11]. The mammary gland is a dynamic tissue that undergoes significant changes throughout a woman's lifetime, especially during pregnancy and following the menopause [12]. The development of the breast is exquisitely sensitive to interactions between the epithelium and the stroma [13]. Stromal changes are required for the establishment of cancer [14]. The formation of vascular stroma in breast carcinoma is a process that involves complex reciprocal interactions among tumour cells, endothelial cells, and stromal cells [15].

Epithelial tumours lose their tissue organisation, become differentiated, and secrete abnormal quantities of ECM in a process that resembles wound healing and that is connected to the invasive and metastatic capacity of the primary tumour [16]. Underlying these events is a process known as epithelial-mesenchymal-transition (EMT) which can be activated during chronic inflammation, for example, in chronic wounds or in cancer tissues. Key cellular alterations that occur during EMT include the loss of cell-cell adhesions and the change in the supporting cellular polarity. These changes affect the cells ability to support collective or individual cell migration [16].

In the EMT processes, epithelial cells gain mesenchymal properties and exhibit reduced intercellular adhesion and increased motility; they can also break through the basal membrane and migrate over long distances owing to profound changes in their cytoskeleton architecture [17]. This review focuses on how breast cancer cells undergo changes that enable them to become motile and break free of the basement membrane in a process that can lead ultimately to metastasis.

## 2. Discussion

### 2.1. The Metastatic Cell

Tumours can be defined by their uncontrolled and invasive growth but their phenotype is regulated in a complex fashion based on interactions of the malignant cells with the tumour stroma including the ECM, the vasculature, and the resident immune system [18]. Initially, a cell within a colony is instructed to disrupt cadherin-based intercellular junctions and acquire a fibroblastoid, motile phenotype, initiating detachment from the primary site. This is enhanced by proteases which digest the basal lamina components and facilitate cell movement through the ECM [19].

### 2.2. Cadherins

Cadherins are a family of adhesion molecules that function in cell recognition, tissue morphogenesis, and tumour suppression [20]. Normal epithelial cells depend on the formation of intercellular adhesion contacts to form a continuous sheet of stratifying cell layers that are attached to one another both horizontally and vertically. Cadherins forming these contacts are abundant in adherens and desmosomal junctions [21] which allow epithelial cells to move together as a sheet during wound repair and angiogenesis. This is known as collective migration and can be found in certain epithelial cell tumours. As epithelial cancer cells progress, the function of cell-cell junction protein is suppressed allowing individual cells to separate and migrate independently. This is known as individual cell migration, which can be subdivided into mesenchymal and amoeboid migration. In mesenchymal migration, the cell forms membrane protrusions at the leading edge and adheres to the substrate in an integrin-dependent manner. They release ECM degrading enzymes to remodel the ECM and form a path. Amoeboid migration occurs when the cell substrate adhesion is weak and is independent of integrin function. Cells move by actin-myosin contractile forces and move within the ECM by squeezing the cell body [22].

Cadherins have a large extracellular domain that binds to the same molecule on an adjacent cell. This results in dimerization of two cadherins on the same cell surface resulting in strong and stable adhesive forces [23]. E- and P-Cadherins are responsible for light cell-to-cell junctions in epithelia known as adherens junctions. N-Cadherin, found mainly in neural tissues and fibroblasts, is less stable and more dynamic [20]. E-Cadherin mediated cell-cell adhesions limit cell motility. The loss of E-Cadherin in epithelial cancers leads to disruption of tight junctions. This disrupts cell adhesion plaques enabling tumour cells to disengage from the primary mass and disseminate by individual cell migration [24]. In the mammary gland, E-Cadherin is cleaved by targeted expression of stromelysin-1: matrix metalloproteinase 9 (MMP 9). This is due to the interaction of N-Cadherin with the FGF receptor on the cell surface that increases transcription of MMP 9 leading to increased cellular invasiveness. In experimental models using mice over expression of E-Cadherin impairs invasiveness of tumour cells [20].

N-Cadherin mediated cell-cell adhesion, in contrast to E-Cadherin, is required for collective cell migration [25]. Normally membrane extensions are suppressed in cells that are in contact with at least two neighbouring cells at the anterior and posterior ends [25]. This results in cell polarity. N-Cadherin mediated cell-cell adhesion allows cells to move in sheets and functions in directional migration and contact inhibition. Upregulated N-Cadherin in breast cancer cells creates a state of dynamic adhesion that allows both attachment and detachment of individual cells from the primary tumour [26]. P-Cadherin is present in the highly mitotic terminal end ducts. P-Cadherin adhesion is important in mammary gland growth control and when disrupted can enhance cell invasion and tumour aggressiveness [27].

### 2.3. Matrix Metalloproteinases

The matrix metalloproteinases (MMPs) are a family of at least 28 zinc dependent endopeptidases. They degrade proteins in the ECM [28] leading to connective tissue dissolution. They are synthesised both by breast cancer cells and the surrounding stromal cells that may interact with each other [29], and it is likely that cancer cells are able to stimulate production by fibroblasts in a paracrine fashion [30]. In the normal breast, ECM remodelling is a prerequisite to ductal progression, and MMP activity facilitates this progression by removing or breeching the basement membrane and stromal matrix [31]. MMPs are implicated in cancer invasion and metastasis [32] and can be subdivided into four groups depending on their substrate specificity and domain organisations.

### 2.4. Rho Family Small GTPases

Once the ECM has been degraded, it paves the way for cells to move within its framework. Reorganisation of cortical actin filaments is regulated by a highly integrated signalling cascade that transduces extracellular stimuli to the actin filaments. This signalling cascade is governed by small GTPases [33]. Rho family GTPases regulate cytoskeletal dynamics by cycling between inactive GDP-bound and active GTP-bound states [34]. When cells are stimulated by growth factors, bound GDP is exchanged for GTP. In the GTP-bound state, Rho family small GTPases are active and interact with specific downstream effectors [22]. Rho GTPase members, including RhoA, Cdc42, and Rac, act as molecular switches on which signalling inputs converge and are transduced into a coordinated array of output pathways leading to actin polymerisation [35] and control of the formation of filopodia (Cdc42), lamellipodia (Rac), and stress fibres (Rho) [22].

P13K (phosphatidylinositol-3 kinase) is activated in response to chemoattractants [36]. This causes asymmetry in the internal signalling of the cell establishing cell polarity which defines the leading edge of the cell [22]. During cell movement, Rac and Cdc42 stimulate the formation of protrusion at the leading edges of the cells and Rho A induces retraction at the tail ends of the cells [33]. Activated Rac and Cdc42 induce reorganisation of the actin cytoskeleton at the leading edge. Localised actin polymerisation at the leading edge pushes the membrane forward generating the locomotive force in migrating cells [22]. RhoA regulates stress fibre formation and focal adhesion assembly that results in actomycin contractility and contraction at the rear of the cell which leads to translocation of the cell body [37].

### 2.5. Filopodia, Lamellipodia, and Invadopodia

Cells migrate forward by extending membrane protrusions [38]. In normal cells, these extensions are used to explore the extracellular space and find their way towards their targets in chemotactic location. Metastatic cells use this in cell migration [39]. There are two types of actin filaments. Branched actin filaments are normally associated with cortical areas and nonbranched actin filaments such as stress fibres that are frequently associated with focal adhesions, adherent junctions, and filopodia [40].

Filopodia are thin cylinders containing tight bundles of long actin filaments which protrude from the cell extending tens of microns from the main cortex. They are oriented in the direction of propulsion. Parallel actin bundles form the core structure packed tightly with noncontractile bundles cross-linked by F-actin binding proteins [41]. Lamellipodia are thin propulsive sheets that dominate the leading edges of fibroblasts and other motile cells. Lamellipodia have characteristic ruffle edges due to the leading edges lifting up from the substrate and moving backwards. Actin filaments in lamellipodia are oriented in a cross-weave pattern between two sets of filaments oriented approximately forty-five degrees to the direction of propulsion [42]. Podosomes are cell substrate adhesion sites which are usually observed in adhesive cells, for example, osteoclasts and macrophages. Podosomes are termed invadopodia in oncogene-transformed cells [22]. Invadopodia have the capacity to degrade the underlying matrix [43]. This distinction between podosomes and invadopodia is obscure. As invadopodia persist longer than podosomes, podosomes are thought to be the precursors of invadopodia [22].

### 2.6. WASPs

The formation of cell protrusions is driven by assembly of actin filaments at the leading edge [38]. During development, dynamic remodelling of the cytoskeleton allows the precise placement and orientation of developing tissues. There are many physiological signals that can trigger changes in cell motility and shape [44]. Although Rac and Cdc42 are known to be essential for cell movement, the downstream molecules involved in actin filament reorganisation are known as the WASP family of proteins [33]. Wiskott-Aldrich syndrome is an X-chromosome linked hereditary disease that is characterised by thrombocytopenia, eczema, and immunodeficiency. The gene that was mutated was isolated and named Wiskott-Aldrich syndrome protein (WASP) [45]. To date, five mammalian WASP family proteins have been identified; WASP, neural WASP (N-WASP), and WAVE (WASP family verprolin-homologous proteins) 1, 2, and 3 [22]. These proteins have been identified as the link between the small GTPases and the actin cytoskeleton [33].

WASP binds to Cdc42 through its GBD/CRIB (GTPase binding domain/Cdc42 and Rac interactive binding domain) [33]. This brings about a conformational change in the WASP protein exposing the VCA (verprolin homology, cofilin homology, and acidic region) enabling G-actin and the Arp2/3 complex to promote actin nucleation and formation of protrusive structures such as filopodia and lamellipodia [36].

### 2.7. The Arp2/3 Complex

The Arp2/3 complex is a stable assembly of seven protein subunits. This complex binds to the sides of actin filaments and is concentrated at the leading edge of motile cells [46]. WASP, N-WASP, and WAVE share common biochemical features that enable them to form a tripartite unit with G-actin and the Arp2/3 complex [35]. The Arp2/3 complex multiplies actin filaments by branching when activated. It is responsible for the dendritic filament array observed in lamellipodia [39] and drives the leading edge of migrating cells. The Arp2/3 activating region of the WASP family protein binds with an active monomer resulting in rapid ATP hydrolysis on Arp2 and the nucleation of a new actin filament on the side of a preexisting filament [47]. Breast cancer with high histological grade has irregular morphology consisting of excessive protrusions due to the assemblages of branched actin filaments [48]. Aberrant expression of Arp2/3 in breast cancer leads to multiple branched actin filaments and leads to an increase in both local progression and poor disease outcome [49].

### 2.8. Cortactin

Cortactin is an actin associated scaffolding protein that regulates cell migration [50]. In the presence of both cortactin and WASP proteins, cortactin facilitates the release of activated WASPs by binding directly to the Arp2/3 complex [40] and activates it to promote nucleation of actin filaments [51] promoting and stabilising branched actin filaments [40]. In breast cancer cells, cortactin is associated with the invadopodium [51], and overexpression of cortactin leads to increased cellular motility and invasiveness [50]. Cortactin was found to be overexpressed in mammary tumours where its expression correlates with increased tumour invasiveness [50].

Research into WASPs and their interactions with other downstream effectors has shown them to be a hugely complex and highly integrated signalling cascade that transduces extracellular stimuli to the actin filaments. There are multiple genes involved in WASP related cell motility which are deregulated in human breast cancers.

### 2.9. Tumour Associated Macrophages (TAMs)

It has been widely shown that breast tumours contain marked leukocytic infiltration that significantly correlates with poor prognosis. The majority of these cells are macrophages and CSF-1 (colony stimulating factor-1) is the main chemoattractant for these cells [52]. TAMs have been shown to promote the migratory phenotype of carcinoma cells, and macrophages are known to form podosomes and invadopodia [43]. In normal tissues, injury results in the local expression of a wide variety of growth factors one of which is CSF-1 [53].

CSF-1 was first identified as a haematopoietic growth factor that stimulates the proliferation, differentiation, and survival of monocytes, macrophages, and their bone progenitors [52]. In damaged tissues, CSF-1 attracts monocytes and stimulates them to differentiate into macrophages in order to mediate an immune response, kill pathogens, stimulate angiogenesis, and affect tissue repair [53]. In the lactating breast epithelium, very high levels of CSF-1 are expressed compared to undetectable levels found in resting breast epithelium [52] as CSF-1 produced by macrophages is implicated in the development of terminal end buds which grow out of the mammary breast ducts [53].

Sapi (2004) [52] reported that 58% of all breast cancers and 85% of invasive breast carcinomas expressed high levels of CSF-1, and this expression was clearly localised in the neoplastic epithelial cells of the tumours as well as in the stromal macrophages. Pollard (2004) [53] suggests that there is a correlation between the expression of CSF-1 and poor prognosis in breast cancers which is due to the availability of CSF-1 to recruit and modulate the behaviour of TAMs. TAMs comigrate with carcinoma cells due to the presence of a CSF-1 EGF paracrine loop; carcinoma cells secrete CSF-1 and express the EGF receptor and macrophages secrete EGF and express the CSF-1 receptor stimulating each other to migrate via EGF activation of the N-WASP signalling pathway and formation of invadopodia [43].

### 2.10. Angiogenesis

Tumours require angiogenesis to grow beyond a certain size and TAMs have been shown to cluster in avascular areas correlating with high levels of angiogenesis and decreased relapse-free and overall survival rates in patients with breast carcinoma [53]. Hypoxia upregulates local cytokines and CSF-1, and these are chemotactic to the TAMs which migrate to the area where they stimulate angiogenesis by expressing VEGF and other growth factors and recruit haematopoietic cells such as mast cells and neutrophils [53].

High levels of macrophage infiltrate in breast tumours correlate with a poor prognosis, and hypoxic tumours have a higher invasive capacity and poorer prognosis than well-oxygenated tumours [54]. Macrophages secrete MMP 9 and their podosomes are capable of directly degrading the pericellular ECM [43]. TAMs also promote tumour invasion by secreting uPA (urokinase plasminogen activator). uPA initiates a proteolytic cascade that results in the conversion of plasminogen to plasmin which mediates proteolysis [53]. uPA further recruits MMPs 9 and 2 enabling the remodelling of collagen type IV, the major constituent of basement membranes [54]. Breast tumour cells are then free to flow out of the ductally constrained tumour mass into the surrounding stroma and subsequently gaining access to the vasculature with the ability to colonise distant sites. High levels of uPA are associated with poor prognosis in breast cancer, and an elevated serum uPA is an established prognostic factor used for determining treatment-based decisions in early breast cancer [53].

## 3. Conclusion

As this review has highlighted, the movement of cells within the breast stroma is a hugely complex and a highly integrated process. There are multiple genes related to cell motility and these become dysregulated in breast cancers allowing cells to break down intercellular adhesive forces, cleave molecules in the ECM, and form motile invadopodia facilitating progression through the stroma. They are chemotactic and recruit other cells to stimulate angiogenesis, growth factors, and proteolysis enabling the breast cancer cells to break free of the basement membrane, gain access to the vasculature, and colonise distant sites.
